# Metal Pollution in the Air and Its Effects on Vulnerable Populations: A Narrative Review

**DOI:** 10.3390/ijms27020720

**Published:** 2026-01-10

**Authors:** Adriana Gonzalez-Villalva, Marcela Rojas-Lemus, Nelly López-Valdez, María Eugenia Cervantes-Valencia, Gabriela Guerrero-Palomo, Brenda Casarrubias-Tabarez, Patricia Bizarro-Nevares, Guadalupe Morales-Ricardes, Isabel García-Peláez, Martha Ustarroz-Cano, José Ángel Salgado-Hernández, Paulina Reséndiz Ramírez, Nancy Villafaña Guillén, Lorena Cevallos, Miranda Teniza, Teresa I. Fortoul

**Affiliations:** 1 Facultad de Medicina, Universidad Nacional Autonoma de Mexico, Mexico City 04510, Mexico; marcelarojaslemus@gmail.com (M.R.-L.); nellylopezvaldez@gmail.com (N.L.-V.); mariacervantes00@gmail.com (M.E.C.-V.); gabygp0902@hotmail.com (G.G.-P.); bcasarrubiast@gmail.com (B.C.-T.); pbizarro@unam.mx (P.B.-N.); gpmr01@ciencias.unam.mx (G.M.-R.); igarciapelaez@gmail.com (I.G.-P.); ustarrozcano@hotmail.com (M.U.-C.); joseangel.jgcs@gmail.com (J.Á.S.-H.); paulinaresendiz5@ciencias.unam.mx (P.R.R.); nancy08@ciencias.unam.mx (N.V.G.); lorena_cevallos@ciencias.unam.mx (L.C.); miranda_rodriguez01@ciencias.unam.mx (M.T.); 2 Departamento de Toxicología, Centro de Investigación y Estudios Avanzados, Instituto Politécnico Nacional, Av. IPN No. 2508, Col. San Pedro Zacatenco, Mexico City 07360, Mexico; 3 Facultad de Ciencias, Universidad Nacional Autonoma de Mexico, Mexico City 04510, Mexico

**Keywords:** metals, air pollution, vulnerable population, pregnancy, prenatal exposure, children and adolescents, occupational exposure, metabolic diseases, older adults

## Abstract

Particulate atmospheric pollution poses a global threat to human health. Metals enter the body through inhalation attached to these particles. Certain vulnerable groups are more susceptible to toxicity because of age, physiological changes, and chronic and metabolic diseases and also workers because of high and cumulative exposure to metals. A narrative review was conducted to examine the effects of key metals—arsenic, cadmium, chromium, copper, lead, mercury, manganese, nickel, vanadium, and zinc—on vulnerable populations, analyzing articles published over the past decade. Some of these metals are essential for humans; however, excessive levels are toxic. Other non-essential metals are highly toxic. Shared mechanisms of toxicity include competing with other minerals, oxidative stress and inflammation, and interacting with proteins and enzymes. Prenatal and childhood exposures are particularly concerning because they can interfere with neurodevelopment and have been associated with epigenetic changes that have long-term effects. Occupational exposure has been studied, but current exposure limits for specific metals appear dangerous, emphasizing the need to revise these standards. Older adults, pregnant women, and individuals with metabolic diseases are among the least studied groups in this review, underscoring the need for more research to understand these populations better and create effective public health policies.

## 1. Introduction

The term “vulnerable population” is used to denote individuals who are at greater risk of adverse outcomes, including but not limited to health complications, exploitation, harm, and discrimination [1]. Susceptibility is considered to be biologically based; it refers to individuals who have characteristics that make them more likely to develop health problems [2]. Vulnerability, in contrast, involves individual susceptibility and a variety of factors that can lead to a health problem, including occupation, economic, social, environmental, or health-related issues [1]. Susceptible individuals are not vulnerable if they are not exposed to air pollutant metals, but the vulnerable population is both susceptible and exposed [2]. This population includes children, adolescents, older adults, disabled people, pregnant women, impoverished individuals, and those with chronic and metabolic diseases, which exhibit a heightened vulnerability to a range of pollutants [1,2,3]. This situation is exemplified by children suffering from asthma, for whom an increase in air pollutants has been demonstrated to augment the incidence of school absenteeism, hospitalization, and exacerbation of chronic respiratory illness. Furthermore, air pollutants have been demonstrated to disrupt ciliary movements, thereby creating a conductive environment for viral infections [4]. An additional example is the population with diabetes mellitus, which is more vulnerable to the toxic effects of environmental pollutants, including metals [5,6]. Occupational workers were considered a vulnerable population in this review due to their high and cumulative exposure to air pollutants, even though other risk factors can increase vulnerability, such as inadequate access to resources that could mitigate health-hazard risks [7].

A wide range of factors have been recognized as contributing to individual vulnerability to certain diseases. These include susceptibility factors such as body mass index (BMI), genetics, epigenetics, age, sex, and prior health conditions. Vulnerability may be associated with factors that increase risk, such as frequency and mode of exposure, prior or ongoing work exposure, and place of residence [8,9]. It is clear that no organ or system is immune to the toxic effects of environmental pollutants, which affect the body through various mechanisms. Among these pollutants, metals are one of the most concerning. Some metals share common mechanisms, such as causing oxidative stress and inflammation, while others tend to target specific organs, enzymes, or metabolic pathways. Research shows that certain genetic variants, such as glutathione S-transferase (GST), cytochrome P450 (CYP), and DNA-repairing enzymes, increase susceptibility to environmental pollutants [10]. Even at very low levels, toxicity remains a concern; metals can enter organisms through pathways used by essential elements, and for most of these metals, the absorption is higher after inhalation [11,12]. This narrative review emphasizes the toxic effects of metals on vulnerable populations, with increased susceptibility due to factors like age, pregnancy, chronic or metabolic diseases, and occupational exposure. It also highlights the need for further research on these groups and for reevaluating safe exposure levels and public health policies.

## 2. Metals in Air Pollution, Sources, and Mechanisms of Toxicity

Metals are present in the Earth’s crust and can be released into the atmosphere through natural sources such as soil erosion and volcanic eruptions. However, human activities like mining, combustion, industrial processes, and battery manufacturing are the primary contributors [13]. In the air, metals can be free or attached to one of the most harmful pollutants: particulate matter (PM). PM consists of carbon particles released during combustion, along with organic or inorganic substances attached to them. The size of these particles influences their health effects. The inhalable fraction of PM includes three sizes: PM10, coarse particles with a diameter between 2.5 and 10 μm that can reach the respiratory tract and extend to the bronchi; PM2.5, or fine particles with a diameter less than 2.5 μm, capable of penetrating the alveoli; and PM0.1, or ultrafine particles with a diameter less than 0.1 μm that can also reach the alveoli and enter the bloodstream [14]. Metals are predominantly found in fine and ultrafine particles and are inhaled into the body. Some populations are exposed to higher concentrations, and occupational exposure remains a concern because levels in certain locations exceed permissible limits, posing health risks [15]. Some of these air pollutant metals are non-essential to humans and highly toxic, such as arsenic (As), cadmium (Cd), lead (Pb), and mercury (Hg). Other metals are essential for some animals, but their essentiality is not universally recognized in humans, including chromium (Cr), vanadium (V), and nickel (Ni). Conversely, essential metals obtained through the diet perform vital functions and are involved in numerous enzymatic reactions in humans. Deficiencies can alter physiology and lead to disease, but at higher levels, these metals can be toxic, as seen with copper (Cu), manganese (Mn), and zinc (Zn) [16]. Most of these metals induce their toxic effects mainly through oxidative stress and inflammation [17,18]. This section reviews the sources, toxicokinetics, and toxicodynamics of the ten most relevant metals associated with air pollution.

Arsenic (As) is a metalloid mainly found in the Earth’s crust along with other minerals. It exists in four oxidation states: −3, 0, +3, and +5 [19]. Human exposure primarily occurs through contaminated water, but it is also present in the air, especially near industries or in occupational settings related to producing pesticides, herbicides, insecticides, wood preservatives, and growth promoters for farm animals, and used in the glass industry, non-ferrous metal alloys, semiconductor manufacturing, mining, and smelting. Ambient air samples have been reported to range from 0.800 to 15.7 ng/m^3^, sometimes exceeding the permissible limits [20,21]. When absorbed orally or through inhalation, arsenic is distributed to the lungs, liver, kidneys, bladder, as well as muscles and nerve tissue. Its excretion mainly occurs via the kidneys through urine. The biotransformation of As includes methylation reactions that can increase its toxicity. Lower doses are linked to oxidative stress, inflammation, and protein alterations, while higher concentrations are associated with genotoxic damage, epigenetic changes, and antiapoptotic and proliferative signaling pathways that contribute to its toxicity and carcinogenicity [22].

Cadmium (Cd) is a heavy metal that has only one oxidation state, +2. It enters the atmosphere through human activities such as burning fossil fuels, excessive fertilizer use, and industrial production of batteries, solar cells, plastics, and paints [23,24]. Cd levels in ambient air (PM2.5) reported in a meta-analysis range from 0.16 ng/m^3^ to 7.98 ng/m^3^, sometimes exceeding permissible limits [25]. Smoking is a major source of Cd, along with occupational exposure. The absorption rate via inhalation varies from 7% to 40%, which is higher than the 5% to 10% absorption rate when taken orally. After inhalation, Cd binds to metallothionein or other metal-transporting proteins and is distributed throughout the bloodstream [26]. It can accumulate in erythrocytes, liver, bones, lungs, pancreas, ovaries, testes, and brain, with a half-life of 10 to 30 years [27,28]. Its excretion is slow and mainly occurs through the kidneys via urine. The primary toxic mechanisms of Cd are oxidative stress and endocrine disruption [29].

Chromium (Cr) is a transition metal found in soil, rocks, and volcanic dust [30,31]. It exists in two stable forms: trivalent Cr (Cr III), which is essential for some animals but not proven to be essential for humans, and hexavalent Cr (Cr VI), which is highly toxic to humans [32]. The main sources of atmospheric release are human activities such as the wood and paper industries [33], leather tanning [34], paints [35], and mining [36], which can also contaminate soil and water. Ambient air concentrations of Cr in urban and polluted areas range from 10 ng/m^3^ to 450 ng/m^3^, exceeding the permissible limits for this metal [11]. Inhalation is of particular concern because Cr is easily absorbed, with peak levels occurring about 6 h after exposure. It is distributed to the lungs, liver, brain, spleen, and heart [37] and can cross the placental barrier [38]. About 60–80% of Cr is excreted through the kidneys (urine), 10% through bile, and the remaining 30% through hair, nails, milk, and sweat [37]. Inside the body, Cr IV can form, and its toxicity stems from its structural similarity to phosphates and sulfates, enabling it to enter cells via ionic channels, where it is then reduced to Cr III. This process generates reactive oxygen species (ROS), causing oxidative stress that damages DNA, lipids, and proteins [18,39].

Copper (Cu) is a transition metal with an atomic number of 29 that is essential for living organisms [40]. In humans, it functions as a cofactor for enzymes, aiding in iron absorption, supporting connective tissue, and regulating lipid metabolism [41]. It is released into the atmosphere mainly through human activities such as mining, foundries, and fuel burning [42]. Cu nanoparticles have also been released into the environment because of agricultural activities and wastewater treatment plants [43]. Cu concentrations in PM2.5 over the past decade in cities across different countries range from 3.11 ng/m^3^ to 54.56 ng/m^3^, with a study reporting increased levels of 1184.88 ng/m^3^ [25]. It is absorbed orally via copper transporters (CTR1) [44]. During inhalation, it may use other transporter channels. In organisms, its oxidation states are Cu^+^ and Cu^2+^ [45]. Accumulation of this metal occurs in the liver, brain, heart, and kidneys [46]. Excretion is mainly via the hepatobiliary system, with a small amount eliminated by the kidneys through urine [47]. Exposure to high concentrations of Cu or prolonged exposure may have toxic effects, mainly through mechanisms like oxidative stress, mitochondrial damage, DNA breaks, and a form of cell death called cuproptosis [48].

Lead (Pb) is a heavy metal of historical significance that remains a major global concern because there are no safe levels for this non-essential metal [49]. Pb occurs naturally in the Earth’s crust and has been used since prehistoric times to make tools, pipes, and weapons. Exposure to Pb mainly happens through ingestion and inhalation [15]. Pb was released into the air for many decades through the burning of leaded gasoline, which has now been banned in most countries [50]. Nowadays, the primary sources of atmospheric Pb are burning lead-containing materials, mining activities, foundries, paints, and battery recycling plants [12,49]. Atmospheric Pb concentrations vary by location, ranging from 0.3 to 1 μg/m^3^ in large cities of developing countries and exceeding 10 μg/m^3^ near foundries [12]. A meta-analysis reports Pb concentrations in PM2.5 ranging from 5.6 ng/m^3^ to 155.96 ng/m^3^ across different countries, with a reported value of 500 ng/m^3^ [25]. After absorption, Pb enters erythrocytes and, in small amounts, is transported via proteins such as transferrin and albumin. Pb is distributed and over 90% accumulates in bones and teeth, but also in the kidneys and liver [51]. Pb in bones can persist for decades, with an estimated half-life of about 30 years [11]. The long half-life of Pb in bones is concerning, especially in conditions that promote bone resorption, such as pregnancy, lactation, or osteoporosis, which can increase blood lead levels (BLLs). The primary route of Pb elimination is through the kidneys via urine [52]. Oxidative stress is the main mechanism by which Pb exerts its toxicity, but other processes include interfering with calcium signaling, mimicking divalent cations, inhibiting specific enzymes, causing genetic damage, and disrupting hormone function [12].

Manganese (Mn) is an essential metal for many organisms, including humans. Its effects are related to enzymes involved in antioxidant defense, digestion, reproduction, immune function, neurological function, and energy production; however, increased concentrations can be toxic [53]. It is the fifth most abundant metal on Earth. It can be released through oil combustion and industries such as battery manufacturing, welding, and metalworking [54]. Its oxidation states are +2 and +3. It is absorbed via divalent metal transporters, and in plasma it binds to transferrin [54]. Mn in the ambient air has not been reported to exceed the permissible limits [54]; however, workers can be exposed to higher levels of Mn. Inhalation of fumes and dust enters the lungs, but it can also enter through the nasal mucosa and reach the olfactory bulb in the nervous system. This metal accumulates in the liver, pancreas, bones, kidneys, and brain. Elimination of Mn is mainly by the liver, via hepatobiliary excretion into the feces, but also through the kidneys in urine, and sweat. Its toxicity mechanisms include oxidative stress, mitochondrial dysfunction, protein misfolding, and dysregulation of autophagy [53].

Mercury (Hg) is a heavy metal and the only metal that is liquid at room temperature. It can be found as an inorganic compound, such as metallic mercury (Hg0), mercurous cation (Hg_2_^2+^), or mercuric cation (Hg^2+^); or in organic form, mainly methylmercury produced by bacteria [55]. The atmosphere is the main medium for the global transport of Hg. Its sources include volcanic activity, erosion, emissions from biomass burning, gold mining, and certain industries [56]. Other sources are tooth amalgams and gold jewelry. This metal can be inhaled and absorbed, leading to accumulation in the brain and kidneys. Hg0 crosses the blood–brain barrier; Hg^2+^ has limited ability to do so but can use cysteine transporters to enter the nervous system; it can also accumulate in the placenta, fetal tissues, and amniotic fluid. Its excretion mainly occurs through the kidneys via urine, with a half-life of 42 days [56]. Hg toxicity depends on the compound and its oxidative state, so organic compounds are absorbed more readily than inorganic ones [15]. Its toxicity results from oxidative stress, mitochondrial dysfunction, and immune–neurological disruption. It interacts with amine, amide, carboxyl, thiol, and sulfhydryl groups of proteins, alters calcium homeostasis, and modifies glutamate regulation. Reports indicate that microtubule inhibition, enzymatic inhibition, genotoxicity, and autoimmune responses may occur [57].

Nickel (Ni) is a transition metal with oxidation states ranging from −1 to +4, most commonly +2. It is extracted from mines, and its malleability and corrosion resistance make it valuable for use in metal alloys employed in the steel, construction, automotive, and renewable energy industries. It is also present in tobacco, nickel–cadmium batteries, coins, jewelry, watches, household and kitchen utensils, and orthodontic and orthopedic devices [58,59]. These industries, along with fossil fuel combustion, are major sources of air pollution and soil and water contamination. Ni concentrations in PM2.5 range from 0.9 ng/m^3^ to 13.39 ng/m^3^, with a maximum of 164.53 ng/m^3^. These levels exceed the allowed limits [25]. Inhalation is the primary route of nickel exposure, with roughly 20% of inhaled nickel absorbed. It enters cells via iron transporters and may also use clathrin-mediated endocytosis. Nickel is mainly excreted through the kidneys via urine and, to a lesser extent, through saliva and sweat [58]. Nickel is cytotoxic, causes DNA damage, and promotes apoptosis [60]. It triggers oxidative stress and activates several signaling pathways, including mitogen-activated protein kinases (MAPKs) [61], phosphoinositide 3-kinase (PI3K) [62], hypoxia-inducible factor 1-alpha (HIF-1) [63], and nuclear factor-kappa B (NF-κB) [64].

Vanadium (V) is a transition metal that can exist in various oxidation states from −1 to +5. Vanadium pentoxide, the most common V compound in the atmosphere, is also among the most toxic [65]. V is considered an essential metal for some species but not for humans. It is used in pigments, batteries, steel alloys, fertilizers, and nutritional supplements. V is mainly released into the air after fossil fuel combustion because it is present in high concentrations in oil [66]. It is estimated that about 70,000 to 210,000 tons of V are emitted into the atmosphere each year [67]. Inhalation is the primary route of absorption [68]. V can enter cells through ion channels, phosphate or sulfate channels, or transferrin transporters and is distributed and accumulates in the heart, brain, liver, and kidneys, with excretion mainly through urine [69]. V can disrupt membrane transport, increase ROS production, and decrease antioxidant levels, leading to oxidative stress and inflammation. Additionally, its similarity to phosphates enables it to interfere with enzymes such as ATPases and phosphatases, thereby affecting signaling pathways like MAPK and JAK/STAT [70,71]. Oxidative stress caused by V can result in DNA damage, apoptosis, mitochondrial damage, and lipid-peroxidation-related membrane injury [67,72].

Zinc (Zn) is a transition metal that naturally occurs in its divalent form. It is an essential metal that is crucial for various enzymes and cellular processes. However, high concentrations of Zn can be toxic. Most Zn is absorbed through oral intake; however, inhaling air pollution is another risk factor for intoxication [73]. Ambient air concentrations of zinc range from 21.2 ng/m^3^ to 35.42 ng/m^3^, but there are no reference values or permissible limits established [25]. It seems that Zn does not accumulate in the long term; therefore, toxicity results from imbalances in Zn regulation: when Zn levels rise, metallothionein levels also increase, promoting its excretion in urine. Nonetheless, metallothionein has a higher affinity for Cu, which can lead to Cu deficiency and explain many toxic effects linked to Zn poisoning [74]. Wilson’s disease is a risk factor for Zn toxicity because its treatment involves Zn supplementation to lower Cu levels in the body [75].

Table 1 summarizes the mechanisms of toxicity and molecular targets of each metal, including shared mechanisms such as oxidative stress, inflammation, nitrosative stress, lipid peroxidation, DNA damage, and mitochondrial dysfunction. It also highlights specific mechanisms, such as enzyme-binding groups and enzyme inhibition.

## 3. Vulnerability Due to Age-Related Susceptibility

Some periods in life are marked by age-related physiological changes that make individuals more susceptible to damage from airborne metal pollutants. Prenatally exposed individuals are developing all the tissues, organs, and systems, so this time is particularly susceptible to damage from toxic agents. The growth and development of organs and systems continue into early life, so children and adolescents are also susceptible. Older adults are among the most vulnerable populations because aging alters organ and system function, and it is challenging to isolate aging-related susceptibility alone, as some of these individuals have other susceptibilities, such as chronic or metabolic diseases, or factors that increase their vulnerability, such as social or economic issues.

### 3.1. Prenatally Exposed Individuals

Most studies on pregnancy have reported effects on the embryo or fetus, as well as the postnatal consequences in children. Pb exposure has been linked to intrauterine growth restriction, low birth weight and length, and an increased risk of preterm birth. Maternal factors such as age, socioeconomic conditions, and educational level have been linked to increases in their vulnerability [81]. It is important to recognize that metals can cross the placental barrier because they can compete with essential metals and ion transporters. There is sufficient evidence connecting preterm birth with Pb exposure and suggestive evidence for Cd and Cr [82]. Although most Cd accumulates in the placenta, prenatal exposure leads to preterm births, low birth weight, and decreased cephalic and thoracic circumference [83,84]. Hg is linked to pregnancy loss, birth defects, or cognitive impairments in children [85]. Methylmercury exposure during pregnancy, even at low doses, can accumulate in the fetus, leading to congenital malformations, low birth weight, and stillbirths [86,87]. Cr is associated with low birth weight, altered development, oxidative stress, and placental insufficiency. One proposed mechanism is that Cr can activate estrogen receptors, thereby inhibiting fetal growth [88,89]. Maternal exposure to V, As, and Pb has been associated with low birth weight, possibly due to impaired maternal thyroid hormone (T3) levels [90]. Also, V has been associated with decreased cephalic and abdominal circumferences and preterm birth, which increases the risk of neonatal deaths [91,92].

Individuals exposed to metals prenatally are at a higher risk of neurodevelopmental impairments, which may result in ADHD or ASD. Some studies have shown links between prenatal exposure to Cd and ADHD as well as cognitive changes [93,94]. Pb prenatal exposure has been associated with ADHD and ASD [95]. Umbilical cord BLL has been linked to changes in cognitive and psychomotor development [81]. Arsenic (As) has been linked to ASD, especially when birth weight is 2500 g or lower [96]. Epidemiological studies suggest that Ni prenatal exposure may contribute to ASD and cerebral palsy [97,98]. In utero exposure to Mn has also been associated with ASD and ADHD, likely due to neurological oxidative damage [54]. Another study linked As, Cd, and Mn to ASD, while Cd and Hg were associated with ADHD, and Cu displayed a U-shaped relationship with ADHD, increasing risk at both low and high levels [99]. V prenatal exposure, with stronger associations in the third trimester of pregnancy, was associated with postnatal mental development index (MDI) in boys, and this appears to be due to V endocrine disruption, along with other well-studied mechanisms, such as oxidative stress and inflammation [100].

The hematological and immune system may experience consequences because of prenatal exposure to metals. Zn exposure may lead to anemia and neutropenia, which affect children’s growth and development [101]. Zn exposure during prenatal and childhood stages has an immunosuppressive effect, as it lowers T lymphocyte levels [102] and increases the risk of respiratory infections [103]. Hg prenatal exposure is associated with harmful immunological effects [104]. In a mouse model, in utero exposure to As impaired lung development, resulting in postnatal changes in respiratory function [105]. Pb prenatal and postnatal inhalation has been linked to a higher risk of asthma in children [106]. Increased levels of Cd in mothers’ urine were related to lower CD4+ and CD8+ lymphocyte counts in their children, potentially predisposing them to autoimmune diseases [107]. Prenatal exposure to Cd is also connected to eczema, food allergies, and asthma [108].

Prenatal exposure to As is associated with physiological and epigenetic changes in both mothers and their offspring, as shown in human and animal studies. These changes include glucose intolerance, impaired insulin secretion, hyperlipidemia, and hepatic steatosis [109,110,111]. All of these are features of metabolic syndrome, and the mechanisms by which As exposure is linked to this condition are not yet fully understood [112]. Additionally, there has been concern about the association between prenatal exposure to As and cancer in later life [21].

Zika virus infection during pregnancy can cause congenital Zika disease, which leads to neurological damage in newborns. It has been observed that high levels of Cu are associated with this disease, as they increase oxidative stress and contribute to astrocyte damage in the nervous system. Authors recommend monitoring essential mineral levels in pregnant women and administering Cu chelation therapy to women at risk of Zika virus infection [113].

One of the most concerning effects of metals that requires further research is the epigenetic changes that could lead to transgenerational inheritance, increasing the risk of neurological, metabolic, and other diseases [114].

### 3.2. Children and Adolescents

Children are more vulnerable to metal atmospheric pollutants partly because they may have a higher inhalation rate than adults, meaning the volume of air inhaled per unit of body weight is greater, depending on activity level [115]. Additionally, the absorption of metals is greater in children, making them more vulnerable to their toxicity, which can lead to severe consequences. Adolescents generally engage in more physical activity and practice sports, which may increase their exposure to air pollutants. They also absorb larger amounts of metals than adults, contributing to their greater vulnerability to the toxic effects of metals [85]. Mn exposure during childhood is so harmful that it is linked to increased infant mortality [116], and very high Pb exposure is linked to seizures, coma, and even death [15].

The lungs are typically the first organs affected by inhaling polluted air. V, Mn, As, and Ni are associated with lung damage. Prenatal and postnatal exposure to As in air-polluted areas has been shown to harm the lungs, causing a restrictive pattern that decreases lung capacity [117,118,119,120]. Ni exposure is linked to altered lung function [121]; in adolescents and children, inhaling it worsens asthma [122], and it has also been associated with atopic dermatitis [123]. V and Mn in air pollution have been reported to impair lung function parameters in children, especially with environmental tobacco smoke exposure [124]. Additionally, areas with high levels of airborne Pb are linked to increased blood lead levels greater than 5 mg/dL in children and a higher prevalence of asthma [13]. Cd exposure through inhalation has been linked to lung function impairment in children as well [107].

Metal air pollutants may impair the hematological and immune system in children and adolescents. Pb has enough evidence that it causes anemia [125] by affecting erythrocyte survival and heme enzyme activity. Additionally, iron deficiency is a risk factor for increased Pb absorption and anemia [126]. High levels of Zn in the blood of children after inhalation exposure are toxic because they may be related to Cu deficiency; both essential metals are inversely related. The main signs of zinc toxicity are microcytic anemia and neutropenia [127].

Neurodevelopment continues during childhood and adolescence, making these populations more vulnerable to the neurotoxic effects of metals [15]. Cd, Mn, Pb, and As are associated with neurodevelopmental impairment [128]. In a Pb review, the ability of Pb to cross the blood–brain barrier and its capacity to accumulate in neurons and glial cells were discussed, along with the fact that most children and adolescents remain asymptomatic but may experience cognitive impairments, learning disorders, memory problems, behavioral issues, and attention deficits [12,15]. Pb exposure in childhood has been linked to aggressive and criminal behaviors in adolescence and adulthood [15]. A recent meta-analysis reported highly suggestive evidence connecting Pb exposure in children with ADHD, as well as suggestive evidence for decreased intelligence quotient (IQ) [129]. Children exposed to low levels of air Pb and high levels of manganese (Mn) showed lower IQ scores [130]. Additionally, there are reports of increased levels of metals such as Hg, Pb, Cr, and V in children with ASD [131,132]. Learning disabilities have been reported after Hg exposure in children [104]. Prenatal and childhood exposure to high concentrations of Mn is associated with low IQ, impaired reading and mathematical skills, and cognitive, behavioral, and motor impairments, which can lead to altered posture [80]. Also, Cd has been linked to intellectual disabilities, as well as cognitive, emotional, and behavioral changes [133,134]. Children aged 8–16 years exposed to elevated air concentrations of Cu near their schools exhibit motor impairments and alterations in the basal ganglia [135].

In adolescents, exposure to metals may be related to the onset of metabolic changes and chronic diseases. High BLL has been associated with sustained high blood pressure, particularly in younger male adolescents, and is associated with greater susceptibility than in adults. Cadmium (Cd) exposure in the same study was associated with stunted growth in male adolescents [85]. Osteoporosis and different types of cancer have been linked to Cd exposure [15]. Even with low BLL, Pb exposure was associated with obesity in adolescents [136], and cumulative exposure to Pb since the prenatal stage is associated with a greater risk of childhood obesity [95]. Chromium (Cr) is an essential metal; however, elevated concentrations, as found in children with family members working in e-waste recycling plants, have been reported to increase body weight and chest circumference, particularly in boys, compared with children with low-level Cr exposure. Higher body weight is associated with obesity and metabolic diseases [137]. Another metal associated with obesity in children and teenagers is Cu, as higher levels of blood Cu increase the odds for overweight and obesity [138]. Additionally, early exposure to environmental Pb in childhood may induce epigenetic changes that, later in life and across generations, lead to metabolic changes and increase the risk of diabetes [139]. High BLLs are associated with hemoglobin A1C, which may predict diabetes risk in the non-diabetic population [140].

V exposure in adolescents is associated with changes in early markers of kidney tubule-interstitial damage [141]. In female adolescents, it is related to changes in breast density through different stages of life that predispose to breast cancer [142]. The association between high levels of V and more severe chronic kidney disease in children has been identified [143]. Additionally, early markers of kidney damage in adolescents are linked to higher urinary levels of V and Cr [141]. Increased Cd absorption in children has been associated with increased creatinine, urea, and proteinuria, which correlate with blood Cd levels [144,145].

Copper overload is rare because the body usually balances absorption and excretion; however, children exposed to high levels of Cu are more susceptible to Cu toxicity, particularly affecting the liver and kidneys. Zinc treatment or chelation may prevent this toxicity [146].

### 3.3. Older Adults

Advanced age is a risk factor for greater susceptibility to the effects of airborne metal pollution. Environmental exposure to air Pb and Cd in older adults has been associated with a higher prevalence of COPD and a greater risk of all-cause mortality in this population [147], and blood levels of Cu have been positively associated with a higher risk of COPD [148]. Exposure to environmental concentrations of Cr, cobalt (Co), and Ni has been associated with alterations in lung function, mainly restrictive ventilatory dysfunction in the elderly [149].

Additionally, BLLs have been linked to an increased risk of frailty in people aged 80 years and older, due to impairments in Frailty Test scores, activities of daily living (ADLs), instrumental activities of daily living (IADLs), functional limitations, and hearing loss [150]. In another study of older adults, blood levels of Cd and Pb, both individually and combined, were associated with poor working memory and reduced antioxidant defenses [151].

Some metals tend to accumulate in specific organs; for example, higher levels of Cr VI have been reported in the cerebrum of older adults, with evidence of oxidative stress and neurodegeneration, as well as in animal models of Cr exposure. The highest levels have been found in the pituitary and temporal lobe [152]. In a Sprague Dawley rat model, exposure to Cr resulted in more pronounced neurotoxic effects in older rats, including decreased social activity, motor function, and spatial memory [153].

Long-term exposure to lead (Pb) is concerning because it accumulates in bones over decades and might be a better marker for Pb exposure than BLL. Cumulative Pb exposure has been linked to accelerated cognitive aging; higher Pb levels in the patella have been associated with lower scores on the Mini-Mental State Examination (MMSE), faster declines in MMSE scores, and decreases in Word List Total and Delayed Recall test scores in adults and the elderly [154]. Bone Pb levels, but not BLL, have been linked to higher visceral adiposity index (VAI), lipid accumulation products (LAPs), and BMI in aging men [155]. Moreover, Pb has been linked to hypertension in older adults, and coexposure to Mn has additive effects on the association with hypertension. The risk further increases in older adults with comorbidities such as diabetes [156]. Additionally, high BLL has a strong correlation with hyperlipidemia in the elderly, as well as among diabetic and hypertensive patients [157]. Hg bone levels have been positively correlated with VAI and LAP, and Cu, Zn, and Mg bone levels have been positively correlated with LAP; all of these metals are related to metabolic changes in aging men [155].

Ni exposure in older adults has been linked to a high risk of coronary heart disease and heart arrest, potentially due to nickel-induced dyslipidemia, increased sensitivity to nickel effects along with cumulative nickel exposure, and age-related cardiovascular vulnerability. Additionally, nickel urinary levels were associated with an increased risk of diabetes and chronic kidney disease (CKD) [158]. Another metal that increases the risk of cardiovascular disease is Pb, even at lower levels. In a rat model, it has been reported to be cardiotoxic, inducing oxidative stress in cardiac tissue, nitric oxide impairment, and impairment of other metals, in this case, lowering Cu levels [159].

In a meta-analysis, Cd exposure was linked to a higher risk of kidney damage in older adults compared to younger individuals. Additionally, in both hypertensive and older individuals, the estimated glomerular filtration rate (eGFR) decreased after Cd exposure [160]. The nephrotoxic effects of Cd reduce its excretion, increasing the risk of metal buildup in a harmful cycle that affects the kidneys and other organs [161]. V exposure in older adults has been reported to raise the prevalence of CKD [162], as well as metabolic syndrome [163] and hypertension [164].

## 4. Vulnerability Due to Pregnancy

Certain metals have been associated with adverse effects during pregnancy, but limited research has examined their toxic impact on pregnant women. Pregnancy involves physiological changes and increased absorption of essential minerals; this can lead to increased absorption and accumulation of toxic metals, and specific conditions may raise the risk of toxicity. Factors such as increased maternal age and minimal weight gain during pregnancy raise susceptibility, along with poverty, low educational level, and smoking, which increase vulnerability in this context [81].

The large amount of iron required during pregnancy can also lead to the accumulation of other metals. Cd exposure in pregnancy tends to accumulate in the placenta. It has been associated with changes in placental angiogenesis, affecting the expression of placental growth factor (PLGF) and vascular endothelial growth factor (VEGF), which can lead to preeclampsia [165]. In mice, placental angiogenesis is also altered after Cd exposure, but the mechanisms are still unclear [166].

Gestational diabetes has been linked to increased levels of Cd in the meconium or urine of pregnant women [88,167]. The known mechanisms involve oxidative stress and cadmium-induced insulin resistance [28]. One study measured blood levels of Cd and Pb during the second trimester of pregnancy and found associations with gestational diabetes and impaired glucose tolerance, with a stronger link to Cd [168]. Conversely, blood levels of Mn are associated with different pregnancy risks: higher levels increase the risk of gestational diabetes [169], while lower levels are linked to preeclampsia [170]. Exposure to PM2.5 particles with higher Pb content has been associated with endothelial dysfunction and increased blood pressure during pregnancy [89].

In pregnant humans and animals and their offspring, As exposure has been associated with epigenetic changes that increase the risk of metabolic diseases and impair glucose metabolism. In pregnant mice that became obese on a high-fat diet and were exposed to As, the livers exhibited morphological changes, including steatosis and infiltration of inflammatory cells. Reports have also documented changes in the expression of proteins involved in lipid synthesis, inflammation, and oxidative stress [109,110,111].

More research is needed, focusing on pregnant women’s health, to understand and, if possible, prevent the effects of pollutant metals and their toxic mechanisms, as well as to consider the environmental factors that lead to health consequences.

## 5. Vulnerability Due to Chronic and Metabolic Diseases

Environmental exposure to particulate matter (PM2.5) in the air, including metals attached to it, can trigger or exacerbate chronic metabolic diseases, increasing the risk of cardiovascular disease or diabetes through oxidative stress and inflammation [171]. Some metals have been correlated to metabolic diseases and altered glucose metabolism. Since Pb, Cd, Hg, and nickel are hyperglycemic metals, exposure to them may increase the risk of metabolic diseases or worsen them [172]. Cd exposure in men who are overweight or obese has shown a positive correlation with the risk of developing prediabetes [173]. Diabetes and Alzheimer’s disease have been linked to copper overload, which causes Cu-mediated oxidative stress that damages vessel walls and increases the risk of cardiovascular diseases. Cooper suggests studying Cu levels in patients with these conditions, and if elevated, chelation therapy may be indicated. Although his review does not associate environmental copper exposure, the authors of this review wanted to include this possibility [146].

Cd and Pb exposure increase the risk of diabetes and kidney diseases [174]. Additionally, in people with diabetes, higher risks of long-term complications like chronic kidney disease have been associated with Pb and Cd levels in blood and urine [175]. Type 2 diabetes (T2D) patients may be more sensitive than the general population to the toxic effects of Pb, and the kidney is one of the most affected organs since BLL has been correlated with kidney injury [176]. Pancreas damage with decreased beta cell development and function has been reported after exposure to Hg, leading to an increased risk of diabetes, along with metabolic, renal, and cardiovascular alterations [15,177].

Higher levels of Cu in the body contribute to dyslipidemia, atherosclerosis, myocardial injury, and arrhythmia due to cell death caused by cuproptosis. Cu transporters and chaperones regulate free Cu levels, so these molecules are involved in the cardiotoxic effect of Cu. People with preexisting cardiovascular diseases may experience worsening, as well as T2D patients and hypertensive patients [44].

People with pulmonary diseases such as COPD are more vulnerable to air pollution and inhalation of metals than healthy individuals. Exacerbations and progression of pulmonary disease have also been studied [178]. People with COPD and smokers are at a greater risk of Cd-related lung function decrease [11]. Another pulmonary disease, cystic fibrosis, is characterized by respiratory involvement resulting from a mutation in the chloride channel, which is associated with bronchial inflammation. In a study, high Cu concentrations were associated with increased oxidative stress and proinflammatory cytokine levels [179].

Cancer can affect overall health, which in turn increases susceptibility to metal air pollution. Some metals are carcinogenic and, in certain cases, are associated with cancer progression, such as As, Cd, Cr, Pb, Hg, and Ni [180]. Lung cancer progression has been linked to Cd exposure, and Ni, Cr, Cd, Hg, Pb, Zn, and Fe stimulate breast cancer progression [180]. Cancer cells in pancreatic adenocarcinoma have high levels of Cu, which are associated with their survival and growth. It has been suggested that Cu deprivation, along with autophagy inhibition, could be a potential treatment because these cancer cells depend on copper [181].

Metabolic and chronic diseases undoubtedly may impair health and overlap with other susceptibilities, so it is important to consider them to understand how metals affect these conditions and to develop educational programs or public policies to lower the risk.

## 6. Vulnerability Due to High and Cumulative Exposure to Metals

People are exposed to various metals through inhalation in specific workplaces, including different industries, so occupational exposure may lead to organ damage that increases susceptibility and vulnerability. It is concerning that some workers lack protective clothing and equipment, as well as proper care, such as removing their clothes before going home and washing their hands, which increases the risk of transporting metals into their homes and potentially affecting family members [182]. There are reports of acute effects after inhaling certain metals, ranging from minor to severe. A review states that the acute effects of inhaling Ni include nausea, vomiting, cough, and shortness of breath. Still, there are also reports of fatal acute outcomes in spray painting workers who died from acute respiratory distress syndrome after inhaling high concentrations of Ni [58]. High-level exposure to Pb is also linked to severe neurotoxicity, including encephalopathy, coma, and death [12,183].

The exposure to chronic inhalation of metals has been related to many toxic effects in diverse organs and systems. Cr VI exposure results in harmful effects on the epithelium, such as the skin and oral and respiratory mucosa, causing chronic ulcers or even destruction of the nasal septum, due to its potent acid and oxidative properties [184,185]. Additionally, inhaled Cr is associated with rhinitis, bronchospasm, and pneumonia. Over time, it has been linked to chronic pulmonary diseases [186,187]. Aerosol or vapors with Ni inhaled by workers lead to rhinitis and asthma, and it has been linked to pulmonary and nasal cancer [17]. Occupational exposure to certain Zn compounds, such as zinc chloride or zinc oxides, may cause smoke fever, which is frequently underdiagnosed. Additionally, chronic exposure has consequences in the lungs, including asthma, eosinophilia, goblet cell hyperplasia, and pulmonary fibrosis [188,189]. A rare but fatal consequence of the inhalation of zinc chloride in military training is acute respiratory distress syndrome (ARDS) [75]. Pb has been associated with asthma and chronic obstructive pulmonary disease (COPD) and their exacerbation [190]. Additionally, higher blood levels of Pb, Cd, Cr, and Hg have been associated with COPD prevalence in coal miners [191].

Chrome plating workers exposed to Cr, Pb, As, and V, even within occupationally permissible limits, showed increased oxidative stress markers in plasma, as well as DNA damage and lymphocyte activation markers such as 2-integrin, ICAM-1, and L-selectin [192]. Workers occupationally exposed to inhaled As face a high risk of leukopenia and anemia, which have been linked to the inhibition of the JAK/STAT pathway and GATA-1, respectively [193]. Workers exposed to Mn exhibit immunological changes, such as reduced complement C3 and T-cell immunoglobulin and mucin-containing protein (TIM-3) markers [194]. Following these findings, a rat model of high-level Mn exposure demonstrated immunosuppression, shown by decreased humoral responses, including lower levels of immunoglobulins M and G, cytokines, and complement C3 [195].

In a study of workers at a battery recycling plant, a correlation was found between years of exposure to Pb and BLL, ALAD activity, and genotoxicity. Additionally, BLL was linked to lipid peroxidation. The discovery of a 50% reduction in DNA repair mechanisms is concerning and underscores the cumulative impact of Pb occupational exposure [196]. Anemia and altered numbers of leucocytes and platelets have been reported in workers occupationally exposed to Pb [12].

Mn has well-documented neurological effects; “manganism”, a condition similar to Parkinsonism, has been reported in workers with occupational exposure, as well as in animal studies of Mn exposure [80]. Cognitive impairment, working memory issues, and visual alterations are reported in Mn-exposed workers [197]. Dentists and miners exposed to Hg may suffer from chronic intoxication manifested by neurological effects such as acrodynia, which is a polyneuropathy with painful extremities and a dark pink color of the skin. Additionally, they often experience tremors and immunological adverse effects [198]. Short-term memory impairment, executive function alterations, and behavioral problems have been reported in workers with higher BLL and tibia Pb concentrations [12].

Workers exposed to V in air can experience eye irritation, green tongue, lung damage, asthma, chemical pneumonitis, skin lesions, and kidney damage [72,199]. Exposure to Mn was linked to podocyte injury in the kidneys in a mouse model and to the risk of CKD in humans exposed to high Mn levels [200]. In a subchronic exposure study with inhaled Ni at 0.75 mg/m^3^, proteinuria and markers of kidney tubular dysfunction were found in women workers but not in men [58].

It is well-known that some metals have genotoxic and carcinogenic effects. Occupationally related cancers are a public health concern that needs attention to prevent them. Regarding inhaled metals, lung cancer is among the most common types. Pb, Cd, Cr, As, and Hg are recognized carcinogenic metals, and their mechanisms of toxicity include oxidative stress, enzyme interactions, increased cell proliferation, and changes in apoptosis [11,201]. The International Agency for Research on Cancer (IARC) classifies these metals as Group 1: Carcinogenic to humans, such as Cd, Cr, As, Ni; Group 2A: Probably carcinogenic to humans, such as Pb; and Group 2B: Possibly carcinogenic to humans, such as V and Hg. The IARC does not consider Mn, Zn, or Cu to have carcinogenic potential [202]. In a meta-analysis of 47 cohorts, it was found that workers exposed to Cr VI have an increased risk of death from lung, larynx, bladder, kidney, testicular, bone, and thyroid cancers; additionally, cement industry workers and tanners exposed to Cr VI have an elevated risk of cancers in the respiratory system, buccal cavity, pharynx, prostate, and stomach [203]. The mechanisms of Cr genotoxicity and carcinogenicity include DNA double-strand breaks and structural and numerical chromosomal alterations [204]. Occupational inhalation exposure has been associated with lung cancer and other cancers, such as pleura, bone, and melanoma of the skin [205]. Nickel exposure is also genotoxic and carcinogenic and is linked to a higher risk of lung cancer [58].

## 7. Materials and Methods

**Figure 1:**
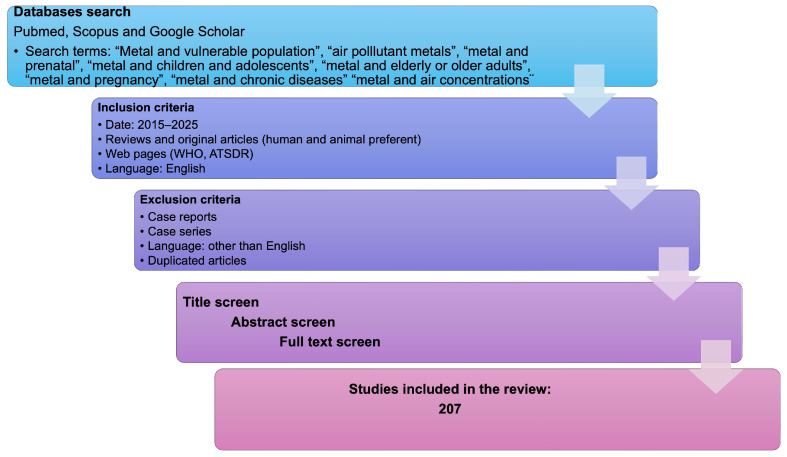
The flowchart illustrates the method used to select articles for this review.

The articles analyzed in this review were searched for in three large databases: PubMed, Scopus, and Google Scholar. The search terms were the name of the metal: arsenic, cadmium, chromium, copper, lead, manganese, mercury, nickel, vanadium, or zinc and the vulnerable population, i.e., “arsenic and pregnancy”. Other essential terms included “air pollutant metals”, “metals and mechanisms of toxicity”, and “metals and air concentrations”. The inclusion criteria were: (1) articles of the last ten years, from 2015 to 2025; (2) reviews and research articles, as well as web pages of regulatory organizations (WHO, ATSDR); (3) language: English; (4) human and animal models preferred. Also, three articles about vulnerability and susceptibility were included. Exclusion criteria were: (1) language other than English and (2) case reports or case series. The first selection was based on the title; the second, on the abstracts; and the finally selected articles were analyzed in full. Duplicated articles were eliminated. The total number of articles included in the review is 207. Figure 1 is a flowchart outlining the steps taken for article selection in this review.

## 8. Conclusions and Future Directions

Metals in environmental air and occupational exposure pose a global threat to human health [9]. Limit values for these metals in environmental air and occupational exposure over an 8-h weight total average are summarized in Table 2, and numerous studies show that these levels are often exceeded [11,15,16].

**Figure 2:**
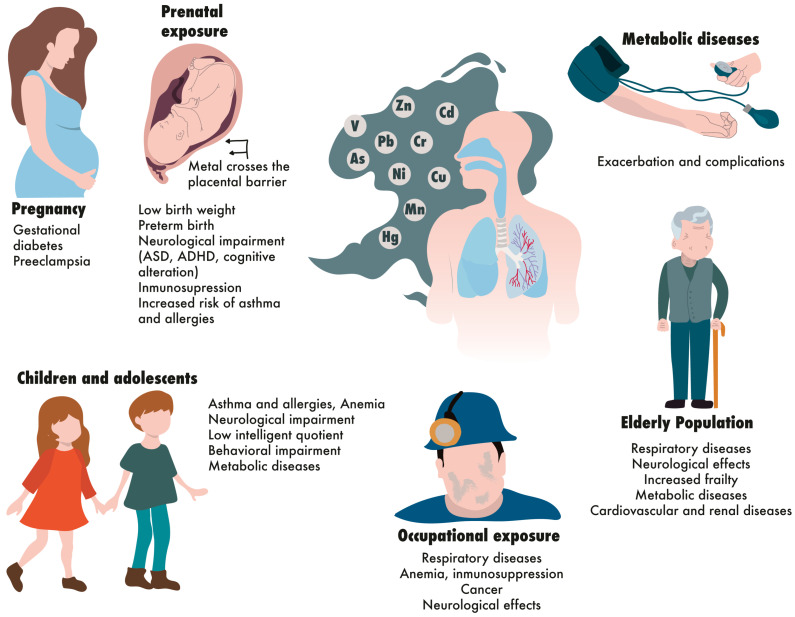
Schematic illustration of how metals in air pollution affect vulnerable populations.

Vulnerable populations are a concern because their characteristics make them more susceptible to harm [1]. Figure 2 illustrates the main toxic effects in these vulnerable populations. Additionally, Table 3 summarizes these effects. In this review, the authors categorized age-related vulnerabilities into groups such as prenatally exposed individuals, children and adolescents, and older adults; vulnerability due to pregnancy; vulnerability due to chronic and metabolic diseases; and vulnerability from high and cumulative exposure related to occupational hazards. The most studied groups found in this review are the individuals prenatally exposed and those with occupational exposure. Pregnant individuals and older adults are among the least studied.

Most research has focused on prenatal exposure and its outcomes, highlighting neurodevelopmental issues that persist into childhood and adolescence, with long-term effects; the strongest link is observed for non-essential metals Pb and Cd [12,129,133]. The consequences of neurodevelopmental alterations, such as ADHD and ASD, cognitive deficits, lower IQ, memory, and behavioral problems, can affect the individual level but also the familial and social levels [54,85,93,94,95,96,98,99,100]. On the other hand, Hg exposure is a significant concern because it is associated with congenital malformations and warrants further research [49,87]. Additionally, there are many reports on the effects of childhood exposure to metals on the respiratory and immunological systems, leading to increased susceptibility to respiratory diseases that persist into adulthood and increased risk of infections and allergies [117,118,119,120,121,122,123,124]. The onset of metabolic and some chronic diseases seemed to be related to prenatal and childhood metal exposure, as evidenced by the large body of evidence for epigenetic changes associated with As, which may be transgenerational, impairing glucose metabolism and increasing the risk of diabetes and metabolic syndrome [28,88,109,110,111,167,168,169]. In this way, more studies of metal-induced epigenetic changes during prenatal exposure are needed. Additionally, there are associations of metal prenatal, childhood, or adolescent exposure with a greater risk of hypertension, obesity, and kidney damage over time that need more research.

Few studies on older adults were found in this review, despite the world’s elderly population increasing. This population is particularly vulnerable due to aging-related declines in organ function and social and economic issues. The risk of all-cause mortality and frailty, as well as lung, neurological, cardiovascular, renal, and metabolic diseases, was found to be associated with exposure to metal air pollutants [149,156,157].

The authors found limited research on the effects in pregnant individuals, and not all the metals included in this review have been studied in this population, even though they have increased susceptibility due to higher absorption, both orally and inhaled, and accumulation of metals, driven by higher requirements for essential metals, such as iron. Respiratory frequency increases during pregnancy, which can also contribute to increased metal uptake. Consequences of metal toxicity, such as gestational diabetes and preeclampsia, may increase the risk of maternal mortality, highlighting a significant problem that could be underdiagnosed [28,88,109,110,111,165,166,167,168,169]. It is important to remember that iron deficiency increases the absorption of other metals. Hence, evaluating iron and supplementing if required are relevant at this stage to prevent the absorption and toxic effects of various metals.

People with chronic and metabolic diseases are especially susceptible to air pollutant metals [15,44,46,171,172,173,174,175,176,177]. The significance for this group is that most of their chronic conditions increase the risk of oxidative stress and inflammation, and metals can exacerbate both in a vicious cycle, potentially raising the risk of complications related to lung diseases, metabolic syndrome, diabetes, hypertension, and cardiovascular and renal diseases. Also, the overlap of other factors that may increase susceptibility, such as older age, malnutrition, and cumulative exposure, should be considered.

The risk of high cumulative exposure, even in apparently healthy middle-aged individuals, has been documented and depends on dose and duration [7]. The consequences of inhaling metals include lung inflammation that may become systemic, affecting various organs and systems. Initial changes increase workers’ susceptibility to subsequent toxic effects. Reports indicate that industrial air often contains metal levels exceeding permissible limits, and even low concentrations that meet permissible limits of certain metals, such as Pb and Cd, can cause adverse effects [29,184,192,205]. There are concerns regarding reports of irreversible neurological and respiratory effects that have resulted in worker deaths. Respiratory diseases and lung cancer are among the most severe outcomes of metal exposure [118,147,189,190,199], along with kidney diseases [160,162].

Additionally, neurological issues, including motor and cognitive effects, have been reported [70,151,152,197]. Long-term studies are needed to fully understand the increased cancer risks to various organs and tissues [22,201,202]. The reevaluation of standard limits for metals in occupational settings, along with the equipment needed to minimize their effects, must be conducted in parallel with evidence and knowledge of their toxic effects to establish safer conditions that preserve workers’ health. Regular monitoring of air metal levels, along with medical examinations and blood or urine testing for workers, must be mandatory and continuous. In the case of Pb, bone levels may help assess cumulative exposure and risk.

Prenatally exposed individuals, children, and adolescents may overlap, as some of the selected studies examine effects over time. Other studies analyze the impact at only one time point related to previous exposure or differ in the age range used to define childhood and adolescence. Despite those differences, the evidence is strong that metals have deleterious effects during these periods, which may alter development and have consequences in adulthood.

A limitation of this review is that it focuses solely on one factor of susceptibility, as is the case with age in older adults, without considering that age frequently overlaps with other health outcomes, such as chronic and metabolic diseases, which may increase susceptibility to metals’ toxic effects. The converse is also true; when chronic or metabolic disease susceptibility is studied, it may overlap with age as another factor increasing vulnerability. Also, in the case of occupational exposure, this review did not consider workers’ age or prior health conditions that may increase the risk of adverse outcomes.

As well as in the other populations, the focus in this review was on pregnancy. Still, pregnant individuals may have other factors that could increase their susceptibility, such as occupational exposure or metabolic or chronic diseases not discussed in this manuscript. Also, vulnerability across all groups may be related to other social or economic factors and should be considered in further research.

The authors believe that future studies should include these other susceptibilities across different populations, as well as analyze disaggregated data based on age, sex, metabolic or chronic diseases, dose, and exposure duration, among other factors. This approach will enrich discussions, enhance understanding of how metals affect this population, and aid in developing improved public policies.

## Figures and Tables

**Table 1:** Metals and their molecular targets and toxicity mechanisms.

Metals	Molecular Targets Toxicity Mechanisms	References
Arsenic	Thiol binding and inhibition of thiol-rich enzymes: pyruvate dehydrogenase, 2-oxoglutarate dehydrogenase, and tyrosine phosphatases. Interaction with zinc finger proteins. Interaction with tubulin (mitotic arrest). Altered MAPKs, NF-κB. Uncoupled oxidative phosphorylation (altered ATP production).	[11,18,21,76]
Cadmium	Metallothionein binding. Increased metallothionein. Interference with zinc-dependent enzymes. Thiol binding and inhibition of thiol-rich enzymes. Mimicking the function and behavior of essential metals (Ca, Zn, Fe), disturbing their homeostasis. Activation of MAPKs, increased *c-fos*, *c-jun*, *c-myc.* Autophagy dysfunction.	[11,18,25]
Chromium	DNA damage. Genomic instability.	[18]
Copper	Interaction with kinases that lead to inhibition of autophagy. LDL oxidation. Altered lipid metabolism. Altered hepatic gene expression. Altered protein–metal interaction.	[77,78,79]
Lead	Mimicking divalent cations (calcium). Inactivation of specific enzymes of heme synthesis (ALAD and ferrochelatase).	[12,76]
Manganese	Activation of enzymes. Mitochondrial dysfunction. Altered homeostasis of other metals (Fe, Ca, and Zn). Dysregulation of glutamate transport. Impairment of dopaminergic function.	[80]
Mercury	Thiol binding Enzyme inhibition. Amine, amide, carboxyl, and sulfhydryl group binding. Glutamate regulation alteration. Calcium homeostasis impairment. Microtubule inhibition. Increased *c-fos* expression	[18,58]
Nickel	Thiol, sulfhydryl binding. Activation of MAPKs, PI3K, HIF-1, and NK-kB signaling pathways.	[61,62,64,76]
Vanadium	Phosphate binding and dysfunction of proteins and enzymes (ATPases and phosphatases). Activation or inhibition of MAPKs and JAK/STAT signaling pathways.	[70,71]
Zinc	Mimicking other metals. Competing for metallothionein with other metals (Cu deficiency).	[74,75]
Arsenic, cadmium, chromium, copper, lead, manganese, mercury, nickel, vanadium, and zinc	ROS production and decrease in antioxidant enzyme levels, leading to oxidative stress and inflammation. Nitrosative stress. Lipid peroxidation. DNA damage. Mitochondrial dysfunction.	[10,12,17,18,22,29,39,48,53,57,61,62,63,64,67,72]

MAPK: Mitogen-activated protein kinase; NF-κB: Nuclear factor-kappa B; ALAD: delta-aminolevulinic acid dehydrogenase, PI3K: Phosphoinositide 3-Kinase; HIF-1: Hypoxia-inducible factor 1-alpha; JAK/STAT: Janus kinase/signal transducers and activators of transcription.

**Table 2:** Limit values for metals in environmental lead and occupational air (8-h average).

Metal	Environmental	Occupational
Arsenic	0.006 μg/m^3^	10 μg/m^3 a,b^
		2 μg/m^3 c^
Cadmium	0.0003 μg/m^3^	10 μg/m^3 a^
		5 μg/m^3 b,c^
Chromium (Cr VI)	0.012 μg/m^3^	500 μg/m^3 a,b,c^
Copper	100 μg/m^3^	Vapors 0.1 mg/m^3 a,b^
		Dust 1 mg/m^3 a,d^
Lead	0.5 μg/m^3^	50 μg/m^3 a,b,c^
Manganese	5 mg/m^3^	20 μg/m^3 a^
		1 mg/m^3 b^
		200 μg/m^3 c^
Mercury	5 ng/m^3^ *	0.025 mg/m^3 a^
		0.1 mg/m^3 b^
		0.05 mg/m^3 c^
Nickel	0.00024 μg/m^3^	0.007 mg/m^3 b^
Vanadium		0.05 mg/m^3 a,c^
	ND	Dust 0.5 mg/m^3 b^
		Vapors 0.1 mg/m^3 b^
Zinc	ND	5 mg/m^3 b^ 2 mg/m^3 a^

^a^ ACGIH, ^b^ OSHA, ^c^ NIOSH, ^d^ ATSDR, * UK health security agency. Environmental air levels [206,207]. ND: not determined.

**Table 3:** Toxic effects of metals in air pollution reported for each vulnerable population.

Vulnerable Population	Metal	Toxic Effect	References
**Age-Related Susceptibility**			
Prenatally exposed individuals	Pb, As, V, Cd, Cr, Hg	Low birth weight Preterm birth	[49,81,82,83,84]
	Hg	Congenital malformations	[49,87]
	Zn, Cd, Hg, As Cd, Pb	Immunosuppression Risk of postnatal respiratory infections Asthma and allergies	[27,102,103,105,106,107,108]
	Cu	Increased risk of congenital Zika (neurological damage)	[113]
	Cd, Pb, As, Ni, V, Mn, Cu, Hg	Neurodevelopmental impairment (ADHD, ASD, cognitive impairment) Lower MDI	[54,85,93,94,95,96,98,99,100]
	As	Epigenetic changes leading to metabolic diseases: glucose intolerance, diabetes, dyslipidemia, liver steatosis. Increased risk of cancer in early life	[21,109,110,111,112]
Children and adolescents	Mn	Increased infant mortality	[116]
	Pb	Seizures, coma, and death	[15]
	Cd	Stunted growth in male adolescents	[85]
	Pb, Cd, V	Osteoporosis Hypertension Increased risk of cancer	[15,52,85,142]
	As, Mn, Ni, Pb, V	Lung damage Asthma Reduced lung capacity Atopic dermatitis	[117,118,119,120,121,122,123,124]
	Pb, Zn	Anemia and neutropenia	[125,126,127]
	Pb, Cr, Cu	Obesity Increased risk of diabetes	[85,127,136,137,138,139,140]
	V, Cr, Cd, Cu	Kidney damage	[141,143,144,145,146]
	Cu	Liver damage	[146]
	Cu	Motor impairment and alteration of basal ganglia	[135]
	As, Pb, Cd, Mn, Hg, Cr, V	Neurodevelopmental impairment: cognitive impairment, learning disorders, memory problems, behavioral issues, attention deficit. Lower IQ scores, ADHD, ASD	[12,15,27,80,128,129,130]
Older adults	Pb, Cd, Cr, Ni	Greater risk of all-cause mortality Lung damage, COPD Increased risk of frailty	[147,148,149,150]
	Pb, Cr	Reduced working memory, MMSE, altered motor function	[151,152,153,154]
	Pb, Mn, Cu, Zn, V	Obesity, hypertension, hyperlipidemia	[155,156,157]
	Ni, Pb	Cardiovascular risk	[155,156,157,158,159,160,161,162,163,164]
	V	Kidney damage	[162]
**Vulnerability due to pregnancy**	Cd, Pb Mn As	Insulin resistance Gestational diabetes Epigenetic changes that increase the risk of metabolic diseases	[28,88,109,110,111,167,168,169]
	Zn	Anemia and neutropenia	[101]
	Cd, Pb	Placental angiogenesis and preeclampsia	[165,166]
**Vulnerability due to chronic and metabolic diseases**	Pb, Cd, Hg, Ni	Increased risk of worsening hyperglycemia, diabetes, metabolic syndrome Diabetes and Alzheimer’s Increased risk of cardiovascular disease	[146,171,172,173]
	Cu	Increased risk of kidney damage Myocardial injury, atherosclerosis, arrythmia, hypertension	[15,44,45,46,146]
**Vulnerability due to high and cumulative exposure (occupational)**	Pb	Encephalopathy, coma, and death	[12,183]
	Ni Zn	Respiratory distress syndrome Smoke fever	[58,75,188,189]
	Cr (VI)	Skin, oral and respiratory epithelium damage, nasal septum necrosis	[184,185]
	Cr, Ni	Rhinitis, bronchitis, dermatitis	[17,187]
	Cd, Cr, Pb, Hg, V	Asthma, COPD	[72,190,191,199]
	Ni, Zn	Pulmonary fibrosis	[17,189]
	Mn, Cr Pb	Parkinsonism Cognitive, short-term memory impairment, behavioral alterations	[12,80,197]
	Hg	Acrodynia (polyneuropathy)	[198]
	As, Pb	Anemia, leukocyte alterations Oxidative stress and DNA damage Lymphocyte activation markers	[12,192,193,194,195,196]
	Mn	Immunosuppression	[195]
	V, Ni, Mn, Pb	Kidney damage	[12,58,72,199,200]
	Pb, Cr, Ni Pb, Cd, Cr, Hg, As	Increased risk of cancer IARC classification 1: As, Cr (VI), Cd, Ni; 2A: Pb; 2B: V, Hg	[58,201,203,204,205]

## Data Availability

No new data were created or analyzed in this study. Data sharing is not applicable to this article.

## Abbreviations

The following abbreviations are used in this manuscript:

ADHD	Attention deficit hyperactivity disorder
ADLs	Activities of daily living
ALAD	Delta-aminolevulinic acid dehydrogenase
ARDS	Acute respiratory distress syndrome
ASD	Autism spectrum disorder
BLL	Blood lead level
BMI	Body mass index
CKD	Chronic kidney disease
COPD	Chronic obstructive pulmonary disease
CTR1	Copper transporters
CYP	Cytochrome P450
eGFR	Estimated glomerular filtration rate
GST	Glutathione S-transferase
HIF-1	Hypoxia-inducible factor 1-alpha
IADLs	Instrumental activities of daily living
IARC	International Agency for Research on Cancer
IQ	Intelligence quotient
JAK/STAT	Janus kinase/signal transducers and activators of transcription
LAPs	Lipid accumulation products
MAPKs	Mitogen-activated protein kinases
MDI	Mental development index
MMSE	Mini-Mental State Examination
NF-κB	Nuclear factor-kappa B
PI3K	Phosphoinositide 3-kinase
PLGF	Placental growth factor
PM	Particulate matter
T2D	Type 2 diabetes
TIM-3	T-cell immunoglobulin and mucin-containing protein
VAI	Visceral adiposity index
VEGF	Vascular endothelial growth factor
